# Application of Zeolite-Based Materials for Chemical Sensing of VOCs

**DOI:** 10.3390/s25051634

**Published:** 2025-03-06

**Authors:** Dusan Stosic, Vladimir Zholobenko

**Affiliations:** 1 Department of Chemistry, Lomonosov Moscow State University, 119234 Moscow, Russia; 2 School of Chemical and Physical Sciences, Keele University, Keele ST5 5BG, UK

**Keywords:** sensors, zeolites, VOC, environmental protection

## Abstract

Considerable levels of pollution produced by urbanization and industrial development have established a need for monitoring the presence of harmful compounds and the assessment of environmental risks to provide a basis for timely reaction and the prevention of disastrous consequences. Chemical sensors offer a reasonable solution; however, the desired properties, such as high sensitivity, selectivity, stability and reliability, ease of fabrication, and cost-effectiveness, are not always easily met. To this end, the incorporation of zeolites in sensor materials has attracted considerable attention. Such hybrid sensor materials exhibit excellent performances due to the unique properties of zeolites, which have been successfully utilized in gas-sensing applications. In this review, we discuss recent findings in the area of the application of zeolites as sensor materials, focusing on the detection of volatile organic compounds and highlighting the role of zeolite frameworks and the proposed mechanisms in the sensing process. Finally, we consider possible future directions for the development of zeolite-based sensor technology, including the application of hierarchical materials, nanosized zeolites, and 2D material–zeolite heterostructures that would fulfill industrial and environmental demands.

## 1. Introduction

The unprecedented global development of the urban, industrial, agricultural, and transport sectors poses a significant threat to the living environment [1]. A huge amount of chemically and physically diverse waste mater is discharged on a daily basis. Various contaminants, such as toxic gases (CO, NO_x_, SO_x_, etc.) [2,3], heavy metals (Cd, Pb, Ni, Cu, Cr, Hg) [4], organic and inorganic chemicals (pesticides, artificial fertilizers, small molecules, endocrine disruptors), volatile organic compounds (VOCs; NH_3_, etc.) [5,6,7], and biological pollutants (viruses, bacteria, etc.) [8], can be found in the air, water, or soil. Environmental pollution has led to significant adverse effects, including vegetation loss, deforestation, soil erosion, elevated levels of harmful chemicals in the atmosphere and food supply, and increased risks of environmental accidents and disruptions to life support systems [9]. Fertilizer-intensive agriculture and the discharge of domestic and industrial wastewater can result in environmental threats such as algal blooms and the suffocation of aquatic wildlife [10]. Furthermore, harmful materials impose serious health issues on humans such as nervous disorders, respiratory failure, kidney injury, heart attacks, asthma and other respiratory complications, etc. [11].

Efficient methods are needed to detect the presence of harmful compounds in the environment in real time before the concentration exceeds hazardous levels. Modern laboratory techniques in analytical chemistry provide high accuracy and sensitivity and enable the adequate determination of even trace amounts of pollutants [12]. However, they can be time-consuming, as a typical analysis may encompass sampling, sample preparation, and calibration prior to the chemical analysis. Chemical sensors offer an alternative solution to sophisticated analytical chemistry equipment [13]. The crucial factors for the selection of a sensor system for environmental monitoring are high sensitivity and selectivity, stability, reliability, size, ease of fabrication, and cost-effectiveness.

Significant efforts have been devoted to developing new sensor technologies, and numerous active materials have been considered, including conducting polymers [14], carbon nanotubes (CNTs) [15], and 2D materials [16]. Semiconductors have been largely considered for gas-sensing applications due to their high sensitivity and low cost. Their application has been hampered by the elevated temperatures needed for their operation, their high energy consumption, and their low selectivity toward analytes [17]. One of the promising methods to improve the stability and selectivity of semiconductor-based sensors is incorporating porous materials, such as zeolites.

In recent years, zeolite materials have attracted significant attention in chemical sensing applications [18]. Zeolites are natural or synthetic microporous crystalline aluminosilicate materials. From their specific and unique structures comes one of the most important concepts for the industrial success of zeolites, that is shape selectivity, which imposes a size-exclusion limit on molecules entering and exiting the zeolite channels by pore confinement effects [19]. The database of the International Zeolite Association (IZA) identifies over 250 synthetic and 40 natural zeolite framework types and provides structural information and crystallographic and spectroscopic data [20].

Overall, zeolites demonstrate a range of characteristics, such as a large surface area, uniform porosity, strong acidity, thermal stability, a high ion exchange capacity, a non-swelling aspect in water, and structural diversity. Furthermore, their properties, e.g., chemical composition and microporous structure, can be modified during synthesis or by post-synthesis modifications. For instance, hierarchical structures have gained increased attention [21]. The small size of the zeolite channels and cavities can impose mass transfer limitations on the transport of bulky molecules. Hierarchical zeolites have been used in order to circumvent these diffusional limitations by coupling the “native” micropores with an auxiliary network of inter- or intracrystalline mesopores. Owing to their many useful features, zeolites, also known as molecular sieves, are extensively utilized as ion exchangers, shape-selective adsorbents, and catalysts [22,23,24]. More recently, a great deal of research has been focused on the development of the potential applications of zeolites as functional materials for battery electrodes [25], super capacitors [26], fuel cells [27], slow-release fertilizers [28], drug delivery [29], etc.

**Figure 1:**
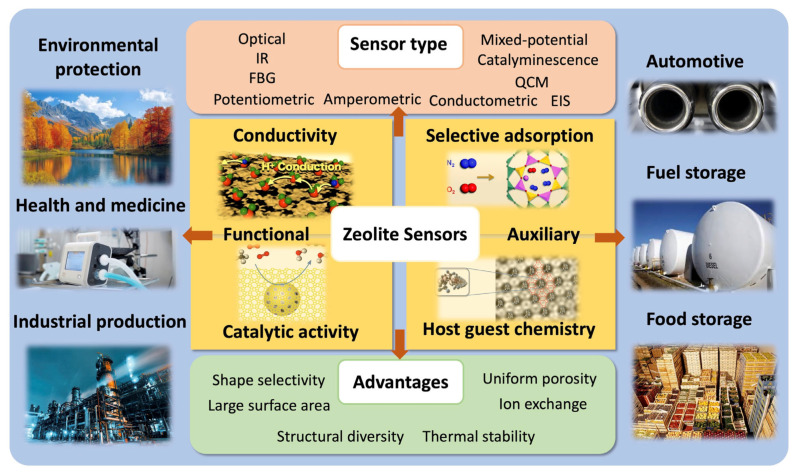
Summary of zeolite use in sensor technologies and related applications.

Zeolites can be utilized in sensor technologies, serving as matrices for other active constituents that include coated systems (a sensitive material coated onto a zeolite and vice versa), as well as host–guest structures with the active material encapsulated within the zeolite framework (Figure 1) [30]. Also, they can be used as functional sensing elements that depend directly on the conductive, adsorptive, or catalytic properties of the zeolite and on its interaction with the surrounding matrix [30]. Zeolites have been successfully utilized in gas-sensing applications [31,32,33], as well as in as electrochemical sensors for the detection of dissolved species [34,35,36].

The present paper will at first summarize the various underlying sensor principles in devices where zeolites are employed as active or auxiliary components. The focus will be on the interaction of the zeolite with the target pollutant, the plausible mechanism, and the application in monitoring volatile organic compounds (VOCs) and humidity. Future perspectives and the possible applications of hierarchical zeolites, nanosized zeolites, and 2D material–zeolite composites in sensing and the benefits that can be gained will be discussed.

## 2. Zeolite Properties and Mechanisms of Interaction with Analyte Molecules

**Figure 2:**
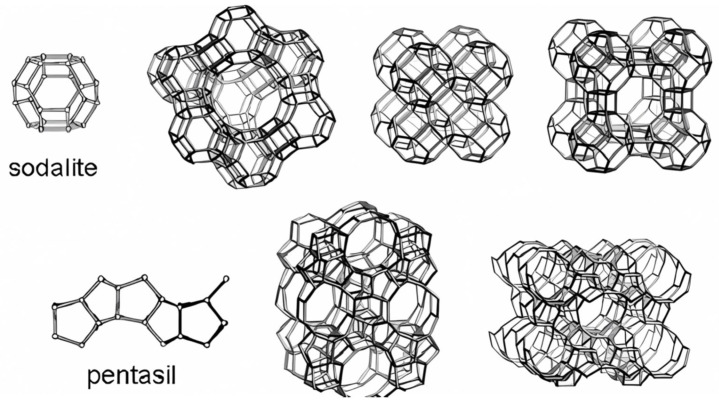
Top—sodalite cage, which is the building block of zeolites X, Y, sodalite, and zeolite A. The lower part of the figure presents the pentasil building unit, together with the structures of ZSM-5 and ZSM-11 zeolites [37].

Zeolites are composed of interconnected TO_4_ tetrahedra as their primary building units (the T is usually Si or Al, but may be the atom of another element including B, Ga, Ge, or Fe), where each oxygen atom is shared between two neighboring tetrahedra to form periodic building units that are further arranged into three-dimensional frameworks with well-defined channel systems and nanosized pores [19]. Figure 2 presents the structures of two common periodic building units (sodalite cages and pentasil chains) and structures of a few typical zeolite framework types [37].

**Figure 3:**
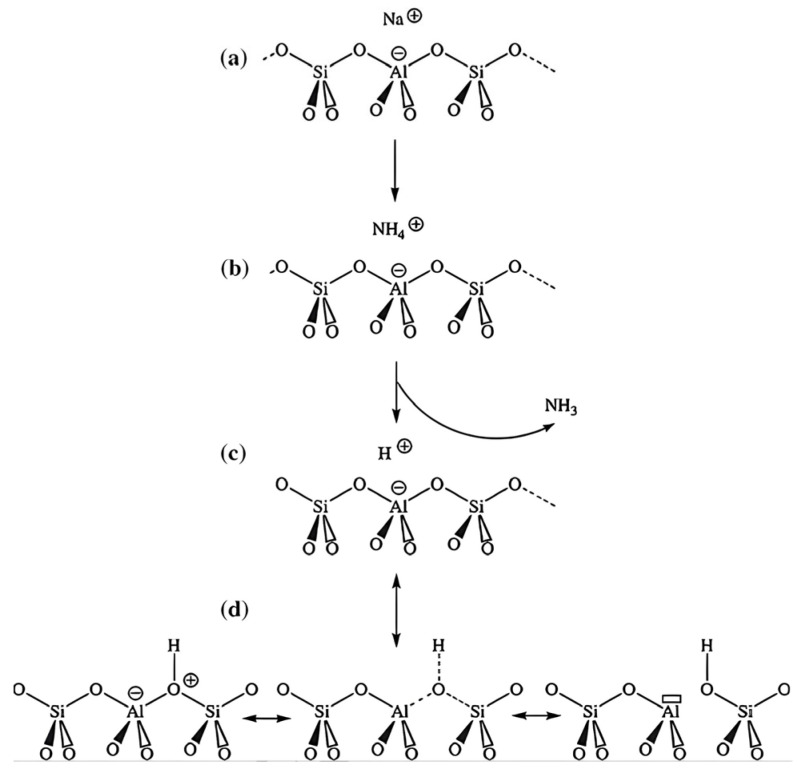
Diagram of the formation of Brønsted and Lewis acid sites in zeolites. (**a**) Charge-balancing cation; (**b**) Na^+^ to NH^+4^ cation exchange; (**c**) from an NH^+4^ cation, an isolated proton (Brønsted acid site) is obtained by calcination; (**d**) Lewis acid site (Al atom with the empty electron orbital, electron acceptor) is generated by a dehydroxylation process [39].

In a zeolite structure, tetrahedral T atoms (Si or Al) are surrounded by four O^2−^ ions, and the network constructed of solely SiO_4_ units is neutral because each O^2−^ ion is shared by two tetrahedral units. The isomorphic substitution of Si^+4^ by Al^+3^ produces a negative charge in the zeolite framework that is compensated for by charge-balancing cations, which are mobile within the zeolite structure, thus bringing about the zeolite’s ion-exchange properties, as charge-balancing cations can be exchanged by other cations, e.g., from aqueous solutions. If cations are exchanged for protons, so-called bridging OH-groups are formed, giving rise to the acidic properties of the zeolites. Both Lewis and Brønsted acid sites can be found in the structure of zeolites [38]. These processes are illustrated in Figure 3 [39].

**Figure 4:**
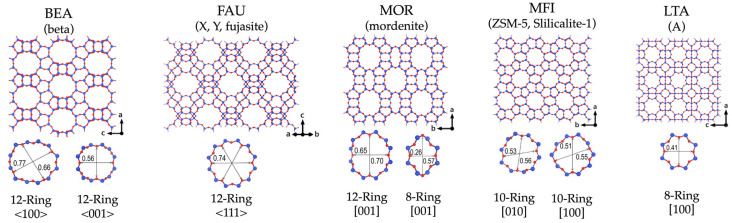
Important zeolite framework types. The three-letter code assigned by the IZA (in bold), the names of the zeolites corresponding to the framework type, the ball–stick atomic model, and the pore windows are provided. Blue circles represent T atoms, and red circles stand for oxygen atoms. The ring sizes (nm) and the directions of the pores are also denoted [20].

The structure of zeolites consists of interconnected rings that determine the size of their pore openings. Depending on the diameter of their largest pore windows, zeolites are categorized into four main types: small-pore zeolites with 8-membered rings, medium-pore zeolites featuring 10-membered rings, large-pore zeolites characterized by 12-membered rings, and extra-large-pore zeolites with more than 12 members in their rings [20]. These channels and pores allow discriminating between molecules with size differences of less than 1 Å. From these features comes the molecular sieving ability of zeolites. Some important framework types and corresponding ring openings are presented in Figure 4.

The role of zeolites in gas-sensing devices can be divided into two major groups depending on their respective function [30], as presented in Figure 1. They can act as the main active element when gas-sensing principles rely directly on the conductive, adsorptive, or catalytic properties of the zeolite material and its interaction with the surrounding medium. In the second group, they are used as auxiliary components, which include coated systems as well as host–guest composites with the active material encapsulated within the zeolite framework. It should be noted here that the distinction between the two groups is not always straightforward, as sensing mechanisms are not always clear and the same zeolite properties could play a crucial role for both groups of sensing devices. Additionally, host–guest assemblies in many cases exhibit physical properties different from either the zeolite hosts or free guest species and can be considered as a new material [24].

### 2.1. Selective Adsorption

The good chemical selectivity and sensitivity of sensors for detecting traces of targeted molecules is vital for the development of reliable detection systems for harmful substances. Among the examined materials, crystalline solids with pores of subnanometric size, such as zeolites, present the highest potential. Close contact between the solid and the adsorbed molecules allows for the development of selective interactions that are suitable to discriminate between the different molecules present in a mixture.

The high surface area of zeolitic materials and their large number of active sites lead to a high adsorption capacity of various molecules from both gas and liquid phases. An increase in the concentration of analyte in a zeolite, as compared to a gas phase, can be several orders of magnitude. For example, Peng et al. [40] estimated the diffusivity of isooctane in classical and hierarchical ZSM-5 zeolites, reporting an increase in concentration in the solid as compared to the gas phase, this being in the order of three orders of magnitude (~440 times). This profound increase in the concentration of the analyte on the surface of the active element or in its vicinity would further have a significant effect on the sensitivity of detection.

Two primary mechanisms can be used as the rationale for a selective interaction between the solid and the analytes [41]. On the one hand, subnanometric-size pores are used as effective molecular sieves, i.e., to exclude molecules with dimensions (characterized by the kinetic diameter) larger than the pore size (Figure 4). This mechanism is able to separate the molecules that are larger than the pore size and those that are smaller but cannot distinguish between molecules of similar dimensions. The other mechanism is related to the selective adsorption that can be achieved with zeolites and other porous materials. Here, the solid either has an intrinsic affinity for a particular chemical species, or this affinity can be obtained by modifying its chemical composition.

**Figure 5:**
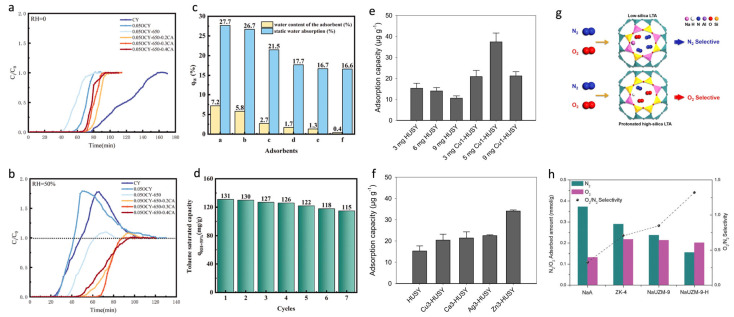
Selective adsorption in zeolites from the gas phase. (**a**) Breakthrough curves of toluene on CY zeolite and modified CY zeolites represent results obtained under (**a**) dry conditions and under (**b**) wet conditions (RH = 50%). (**c**) Static water adsorption capacity (qw) of CY zeolite and modified CY materials (CY—a, 0.05OCY—b, 0.05OCY-650—c, 0.05OCY-650-0.2CA—d, 0.05OCY-650-0.3CA—e, 0.05OCY-650-0.4CA—f), (**d**) seven adsorption–desorption cycles of 0.05OCY-650-0.3CA zeolite under RH = 50%. Reproduced with permission [44]. Copyright 2024, Elsevier Inc. Adsorption capacity obtained (**e**) by varying mass of adsorbent, (**f**) by varying nature of cation in zeolite in series of USY zeolites. Reproduced with permission [45]. Copyright 2022, Elsevier Inc. (**g**) Schematic illustration of O_2_ and N_2_ adsorption processes on low-silica LTA and protonated high-silica LTA. (**h**) N_2_/O_2_ uptake and selectivity of LTA and NaUZM-9-H samples at 25 °C and 100 kPa [46]. Copyright 2020. Royal Society of Chemistry.

The affinity of a zeolite for the adsorption of different molecules is a function of its chemical composition. The Si/Al ratio of a given zeolite is the main factor determining its hydrophilic/hydrophobic character [42]. Silicalite-1 (the Al-free MFI zeolite) is hydrophobic, whereas the hydrophilicity of ZSM-5 (the Al-containing MFI zeolite) increases with an increasing Al content. The affinity of a zeolite for polar molecules follows its hydrophilicity and can be enhanced by increasing the Al content of the zeolite [43]. Xu et al. described the preparation and modification of highly hydrophobic USY zeolite with improved VOC adsorption performance under humid conditions with a 4.2 times higher toluene adsorption capacity (Figure 5a–d) [44]. Furthermore, the incorporation of specific cations, by using the ion-exchange capability of zeolites, has often been proposed as a way to tailor the affinity of a zeolite towards specific molecules. Indeed, in a work by Becker et al., the authors tested the formaldehyde adsorption capacity over cation-exchanged Y zeolites (Figure 5e,f) [45]. It was demonstrated that a higher adsorption capacity was achieved for all the exchanged cations. The best performance was recorded in the case of Zn3–HUSY, which exhibited a 2.5 higher adsorption capacity with respect to pristine HUSY. Liu et al. tested the adsorption capacity of N_2_ and O_2_ on LTA zeolite with a change in its Si/Al ratio (Figure 5g,h) [46]. The results showed that with an increase in the Si/Al ratio and a decrease in the amount of Na^+^, the protonated high-silica LTA zeolite changes from being a N_2_–selective sorbent to an O_2_–selective sorbent.

### 2.2. Conductivity

**Figure 6:**
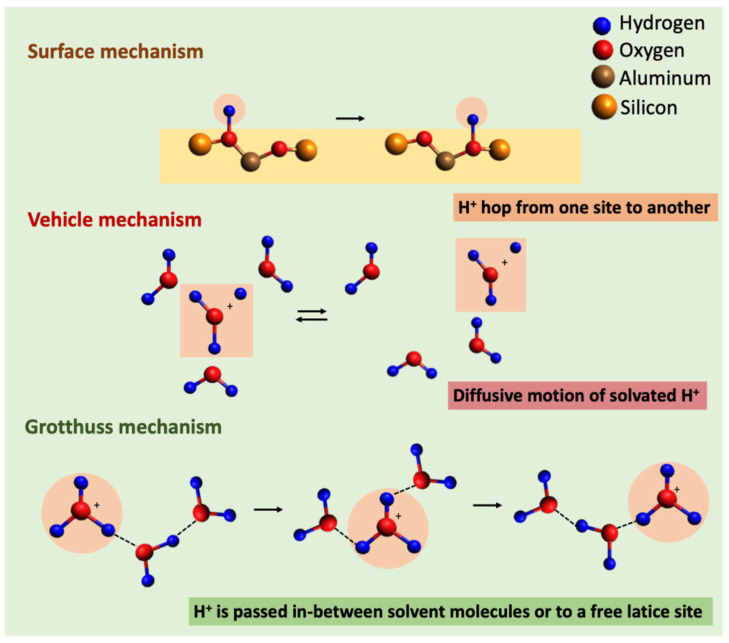
Models of ionic conductivity in zeolites.

Zeolites have a large band gap of several eV, and as a consequence no electric conductivity is usually observed [47]. However, due to the presence of mobile cations in the framework structure that can hop from one binding site to another, they exhibit ionic conductivity (Figure 6). H-form zeolites are protonic conductors due to the mobility of protons, which compensate for the negative charge of the [AlO_4_]^−^ tetrahedra by means of bridging Si-OH-Al groups. If H^+^ is exchanged for another cation, such as Na^+^, then the zeolite is considered a Na conductor. The thermal activation energy of conduction and the conductivity depend on the nature of the mobile cation. In the dehydrated state, the highest conductivity and lowest activation energies were observed for Na^+^-exchanged zeolites [48]. Furthermore, the probability of cation hopping depends on the distance between an occupied and the next unoccupied lattice site and therefore on the Si/Al ratio in the zeolite framework [49]. Schaf et al. studied the influence of water vapor on zeolite conductivity by impedance spectroscopy for HEU, STI, and PHI single-crystal structures in a hydrated state at equilibrium water content. Correlations between the channel geometry and occupation of cation sites were found, showing that the zeolite structure should also be taken into account [50].

The presence of solvate molecules such as H_2_O or NH_3_ in concentrations of above 100 ppm_v_ usually leads to an increase in cation mobility [51]. This increase can be caused by Grotthuss or by vehicle mechanisms, depending on the partial pressure of the solvate molecule (Figure 6). The vehicle mechanism is based on the diffusive motion of solvated protons, namely H_3_O^+^ or NH_4_^+^. In the Grotthuss mechanism, if a proton is bonded to a solvate molecule, it can be transferred to another solvate molecule or to a free framework site via the rotational motion of the solvent molecule. This mechanism requires a sufficient number of solvating molecules between the [AlO_4_]^−^ tetrahedra. Chen et al. provided a more detailed description of how the conductivity mechanism depends on the partial pressure of water in the case of the AlPO_4_-5 zeolite [52]. At a very low humidity, chemisorbed water molecules will be dissociated on the surface, and OH^−^ will associate with Al^3+^, whereas a proton will form an hydroxyl group with surface oxygen. H^+^ from the hydroxyl group has high mobility, and conduction is achieved by protons hopping from site to site across the surface. The further adsorption of water hydrogen from hydroxyl groups forms H_3_O^+^ that is more stable, and the vehicle transport mechanism is considered dominant. Once the chemisorbed layer is formed, further exposure to humidity does not have an effect. Additional water molecules are physiosorbed upon the hydroxyl layer by double hydrogen bonds with the oxygen atom of the H_2_O. However, the surface coverage is still not complete. A further increase in RH leads to the formation of a continuous physisorbed layer. The subsequent physical layers form a liquid-like network of hydrogen-bonded water molecules, in which mobile protons play a primary role as carriers. The local electric field in the chemisorbed layer promotes the dissociation of physiosorbed water molecules, resulting in the formation of H_3_O^+^ and OH^−^ ions. The Grotthuss mechanism stipulates that the charge transport process unfolds when a proton is released by H_3_O^+^ and accepted by a neighboring water molecule, which subsequently releases another proton. This process results in a substantial increase in conductivity.

It should be noted that in a recent article, Wang et al. proposed that Na-type ZSM-5 zeolites can be regarded as ultrawide direct band gap semiconductors [53]. The authors of the study posited that an increase in charge-compensating Na^+^ cations leads to a decrease in the band gap and an effect on its density of states, resulting in a shift of the Fermi level in the direction of the conduction band. Furthermore, the study demonstrated that single-crystalline nanosized Na-ZSM-5 (Si/Al = 9.8) exhibits efficient electron conduction, with a conductivity of up to 10^−7^ S cm^−1^ at 300 °C.

The underlying charge transport mechanism of electrically conductive zeolites is ambiguous, and further work is necessary to clarify it under different experimental conditions (temperature, atmosphere, adsorbed molecules), various framework types, and charge-balancing cations.

### 2.3. Catalytic Activity

Zeolites are among the most used heterogeneous catalysts by the chemical industry. Their catalytic activity and selectivity can be controlled and tuned for a particular process by adjusting the amount of aluminum atoms and hence the amount and strength of active sites, the accessibility of the centers for a molecule of a particular size, and functionalization by dispersing different catalytic materials on to their surface (noble metals, metal oxides, etc.). The accessibility of active sites can be changed by the utilization of standard zeolites with different pore openings (Figure 4) or by the synthesis of hierarchical zeolites that have auxiliary mesopores added to their microporous structure [21]. All this provides a high degree of selectivity that is one of the crucial factors for the design of sensors with very low cross-interference.

Zeng et al. [32] demonstrated the effective use of catalytic activity to improve the sensor response to specific analytes. By incorporating an HZSM-5 zeolite film, the Pd-loaded WO₃ sensor exhibited high sensitivity to CO, with a response of about 10 at 100 ppm CO. Notably, when the HZSM-5 was modified with Pt, a significant enhancement in the sensor response to methanol was achieved. The response was nearly 7 and 15 times higher than that for Pd-WO_3_ and WO_3_, respectively. It was proposed that such an enhanced sensor response to methanol with Pt/HZSM-5 zeolite films may have been a result of the catalytic effect of the zeolite films, which catalytically transformed methanol into an intermediate product that the underlying Pd-WO_3_ is more sensitive to. In addition, the sensor displayed high sensitivity to methanol at high CO concentrations. The authors suggested that the Pt-modified HZSM-5 film catalyzed the conversion of CO, leading to a p-type response to CO.

Mixed-potential sensors belong to the group of potentiometric gas detectors. The device utilizes a solid electrolyte, accompanied by two electrodes that exhibit dissimilar catalytic activities. The voltage output is derived from the competing reactions taking place at each of the electrodes. The distinct catalytic activities of the electrodes result in the establishment of disparate mixed potentials at each electrode, thereby leading to the detection of a potential difference across the device [30].

The cataluminescence effect has been demonstrated as a viable method for the development of optical sensor components. For instance, Yang and colleagues developed devices based on Na FAU or Na/Cs FAU zeolites for the purpose of detecting acetaldehyde selectively [54]. The selectivity exhibited by these sensors was attributed to the zeolite’s pore size and steric effects in relation to the homologous series. The study further established that the nature of the charge-balancing ions plays a pivotal role in determining the selectivity of detection, given its effect on the acidic and catalytic properties of the zeolite. This concept has also found applications in the development of sensors for n-hexane detection [55].

### 2.4. Host–Guest Chemistry

Host–guest chemistry was originally developed as a branch of supramolecular chemistry, in which a host molecule binds a so-called guest molecule or ion. The guest can be fully encapsulated or have free access to the pore openings of the host. The binding energies in the host–guest complexes are usually much lower than those of covalent chemical bonds. The host should have cavities or channels with molecular dimensions and with concave surfaces, as opposed to the convex surface of the guest [56]. Zeolites with different pore widths and architectures of intracrystalline voids have been extensively used as hosts for a variety of materials [56]. Several studies have documented chemical sensors that utilize the intrazeolitic space as a host for guest species, enabling specific interactions with target analytes.

For example, confining single polymer chains in the channels of host zeolites gives an opportunity to design novel hybrid polymer/host zeolite materials with a unique combination of exceptional physical properties, such as the huge surface area of the host material joined with quantum confinement of the π-conjugated polymer. Alabrase et al. studied the polymerization of liquid PhA (phenylacetylene) in the channels of VPI-5 aluminophosphate [57]. Materials were subsequently deposited on QCM, and promising sensing performances to water and butanol vapors, attributed to the finely tuned nanostructure of the composites, were obtained. Furthermore, conjugated polymers can be incorporated inside the zeolite structure for the fabrication of sensors that exploit the conductive properties of such materials.

## 3. Sensing Techniques

### 3.1. Detection of Resonant and Mechanical Changes

Quartz crystal microbalance (QCM) and silicon microcantilever devices are produced with a transducing material (the sensor platform) coated with a layer that is able to recognize the desired target [58,59]. When the layer is made of a zeolite that is designed for the selective adsorption of a desired analyte, these devices can be used as very sensitive sensors. The detection process is based on the changes in the properties of an acoustic wave traveling through the sensor. The transducing material has a characteristic resonance frequency, when an AC potential difference is applied to the quartz crystal, that is sensitive to different external parameters, such as mass perturbations, changing this frequency when the active layer adsorbs the analyte. This change can be directly translated into an analyte mass change, usually with a detection limit on the order of 10^−9^ g cm^2^. It should be noted that although a piezoelectric microcantilever-based transducer uses a similar approach to a QCM transducer, it consists of a cantilever paddle that is anchored at one end to a substrate, with the other end modified with a sensing material and free to vibrate. On the other hand, in the QCM configuration, the piezoelectrical layer is sandwiched between the two electrodes. For additional insights into the application of quartz crystal microbalances in sensing, refer to the comprehensive review by Vashist and Vashist [60]. Furthermore, an in-depth discussion of microresonator theory can be found in the seminal work by Schmidt et al. [61].

Magnetoelastic (ME) acoustic wave (AW) transducers are composed of an amorphous ferromagnetic ribbon coated with a sensing film. The detection mechanism relies on changes in the acoustic wave properties as they propagate through the sensor in response to external perturbations. In this method, a distinct magnetoelastic resonance frequency is displayed when it is under an alternating magnetic field. This resonant frequency changes as the material is subjected to perturbations. Sensors based on the coupling effect between the mechanical and magnetic properties of ME platforms have an advantage in comparison to alternative technologies due to their low cost and wireless detection capability. More information is available in a review on the use of magnetoelastic transducers for sensing applications [62].

### 3.2. Spectroscopic Techniques

Upon the adsorption of analytes, zeolites’ optical properties are changed, and their adsorption capacity can significantly increase the optical response to the detected molecules. Technological advancements in microfabrication techniques and the synthesis of dissimilar materials have led to the development of compact, high-efficiency, light sources, detectors, and optical modulators responsive to small external stimuli [63]. Light interacts with matter in a variety of ways, including refraction, absorption, fluorescence, and scattering. Alterations in the concentration and type of matter in the environment influence these interactions, allowing optical devices to be used as sensors. Progress in the development of optical devices has contributed to the creation of transducers in highly sensitive sensors that convert small changes in the concentration and type of matter into large electrical signals.

Infrared (IR) spectroscopy is a widely utilized analytical technique for identifying and characterizing molecular structures. The IR spectra, defined by the position, shape, and intensity of absorption bands, are influenced by the nature and concentration of molecules interacting with the sensing material. Detection in IR spectroscopy is typically conducted in either transmission or reflection mode [12].

In transmission, IR radiation passes through the sensing material, and the spectrum is recorded at regular intervals. Changes in the IR spectrum resulting from athe dsorption of IR-active species allow both a qualitative and quantitative analysis of target analytes [12]. In infrared reflectance mode, IR light is reflected from a surface coated with the sensing material. The substrate, typically an optical material with a higher refractive index (e.g., a silicon wafer), allows multiple reflections between its outer surface and the inner surface in contact with the sensing material. During each reflection, an evanescent wave passes through the sample. By monitoring the evanescent wave, the energy absorbed at each frequency can be measured, providing detailed spectral information [12].

Fiber Bragg gratings (FBGs) are one of the most popular sensing devices in fiber optics, and they can detect strain and temperature variations [63]. They can be divided into long-period and short-period gratings. The period is defined as the separation between two consecutive fringes of the same refractive index. Long-period gratings (LPGs) typically have periods in the below millimeter range. They work by coupling forward-propagating light of certain wavelengths into forward-coupled cladding modes. The period of the Bragg grating determines the wavelength coupling. However, absorption and scattering losses often reduce the power of these forward-coupled modes. The FBG chemical sensor works on the principle of measuring the axial strain of the fiber resulting from an environmental chemical effect caused by the use of a coating on the fiber that deforms in the presence of a chemical compound. As the FBG expands or contracts, a shift in the reflected wavelength is observed, caused by changes in the spectral pattern of the reflected light. The wavelength shift is monitored by an interrogator, and a quantitative analysis of actual ambient concentrations can be performed using an appropriate calibration protocol [64].

Luminescence-based sensors detect changes in the fluorescence or phosphorescence emission spectrum of a sensing element as a result of interaction with an analyte. Certain molecules, such as O_2_, can suppress the luminescence of the sensing material. The degree of change in luminescence intensity, or wavelength shift, correlates with analyte concentration [65].

Optical sensing can also be accomplished by the incorporation of reactive dyes in zeolite channels and cavities. The resulting color changes induced by the presence of an analyte can be monitored by UV–visible spectroscopy or even visually [66].

### 3.3. Electrochemical Sensing Techniques

There are various electrochemical detection methods, and these include the monitoring of current (amperometric and voltametric), of a potential or charge accumulation (potentiometric), of the conductive properties of a medium (conductometric) between electrodes, and impedance (both resistance and reactance, impedimetric) [67].

Voltametric methods are a group of electroanalytical techniques that operate at a controlled variable potential, where the current is measured in relation to the applied potential. The instrumentation used to perform voltammetry typically consists of a three-electrode system (working, reference, and counter/auxiliary electrodes) known as an electrochemical cell. The operating principle is to detect the anodic/cathodic current generated by the oxidation/reduction of an electroactive species at a selected potential range. By scanning (increasing/decreasing) the potential in a well-defined range where the reduction or oxidation of the analyte is occurring, a resulting current as a function of the potential curve, the so-called voltammogram, is obtained. The voltammogram reflects the electrochemical behavior of the redox species participating in the oxidation or reduction process and can be used for quantitative and qualitative analysis [68].

Potentiometry passively measures the potential difference between the working and reference electrodes, with little effect on the solution. The potential can then be correlated to the concentration of the measured analytes [69].

Amperometry is a technique that involves the measurement of the current generated by the electrochemical oxidation or reduction of an electroactive species at a constant potential. The steady-state current is directly proportional to the analyte concentration. A three-electrode system is the most frequently employed arrangement for these measurements. The working electrode is held at an optimal constant potential relative to the reference electrode [70].

Conductometric gas sensors are solid-state electronic devices in which the surface adsorption of an analyte is directly transformed into a change in the device’s conductance, which is proportional to the concentration of the analyte. The majority of contemporary conductometric gas sensors are based on two primary classes of chemiresistive materials: semiconductor metal oxides and non-oxide materials (e.g., polymers, carbon nanotubes, and two-dimensional (2D) materials) [71].

Impedimetric sensors work by measuring the changes in charge conductance and capacitance at the sensor surface as the analyte is adsorbed. Electrochemical impedance spectroscopy (EIS) is used to measure the impedance changes that occur as the concentration of the analyte changes. The impedance of a system is analyzed by providing a voltage signal of small magnitude at varying frequencies. EIS provides several advantages over the other electrochemical techniques based on the fact that it is a steady-state technique, it relies on small signal analysis, and it is possible to examine signal relaxations over a very wide range of applied frequencies, from less than 1 mHz to greater than 1 MHz. More detailed information can be found in a review by He et al. [72].

## 4. Applications in Gas Sensing

### 4.1. Zeolites as Sensors for Water Detection

The measurement and control of humidity is of great significance in the fields of health and medicine, industrial and agricultural production, food and fuel storage, meteorology, and environmental protection. Although water vapor is not a pollutant, improper levels of humidity can have negative effects on transpiration during plant growth and development, too high or too low a humidity will have an adverse influence on human comfort, and an inappropriate humidity can lead to various problems in industrial and agricultural production. The monitoring of water vapor concentrations in exhaust gas is important for determining the extent of fuel combustion and proper performance of engines. The discussion here is limited to the instances of zeolite-based systems utilized for the detection of both water and VOCs.

**Figure 7:**
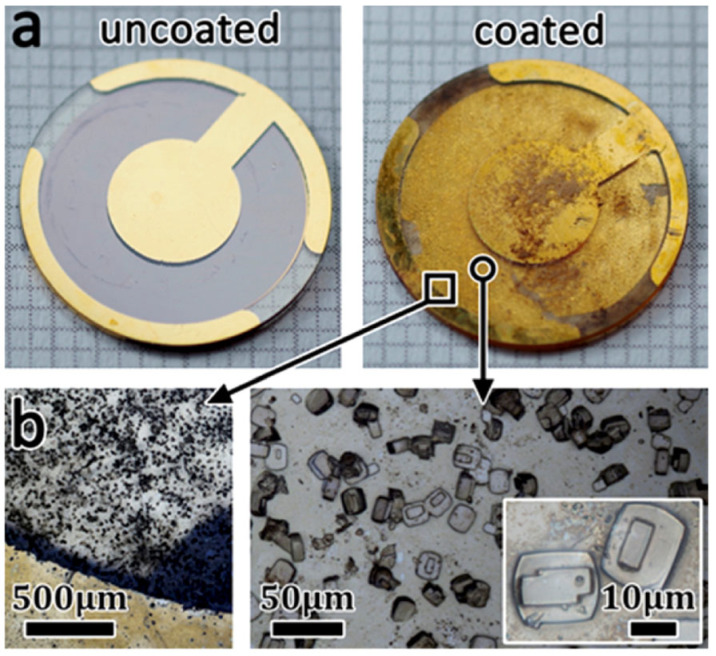
Langatate crystal microbalance sensor coated with ZSM-5. (**a**) Photographs of the uncoated (**left**) and coated sensor (**right**). (**b**) Light microscopy images of the coated sensor [75].

**Figure 8:**
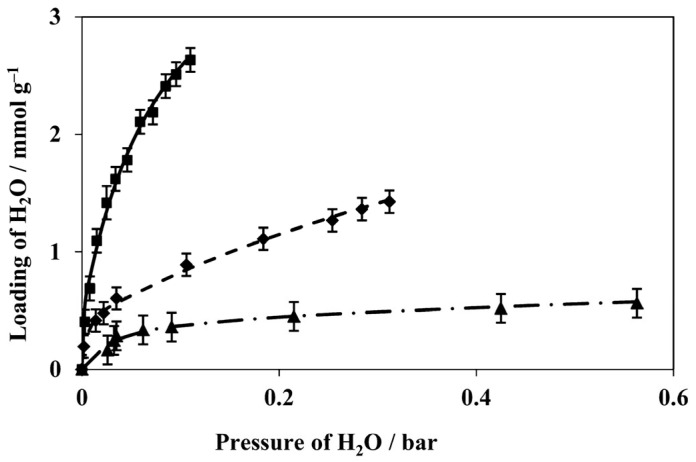
Adsorption isotherms for water in H-ZSM-5 coated on langatate crystal microbalance sensor at 50 (■), 85 (⧫), and 120 °C (▲) [75].

Humidity sensors based on LTA- and BEA-type zeolite films in QCMs prepared using secondary growth on a precursor seed layer were described in ref. [73,74]. Both zeolites showed good thermal stability and exhibited reproducible responses during long-term experiments for the detection of low water concentrations. Response and recovery times were faster for the BEA sensor, which was rationalized on the basis of its larger pore channel diameter (as can be seen in Figure 4) and higher hydrophobicity due to a higher Si/Al ratio, although surprisingly, zeolite BEA showed a greater adsorption capacity for water at low concentrations. It was demonstrated that alkanes would not adsorb on zeolite LTA, whereas the zeolite BEA-based sensor exhibited more interference from these hydrocarbons. Vilaseca et al. developed a sensing system based on commercial QCM sensors modified with AlPO_4_-18 coatings [41]. The best results were obtained with micron-size crystals. Such devices were found to be sensitive to water. On the other hand, their affinity towards non-polar molecules was low, thus enabling the sensing of water in the presence of hydrocarbons. In order to overcome the temperature limitation of a conventional QCM in working temperatures under 80 °C, Ding and co-workers successfully used a langatate crystal microbalance (LCM) to measure the high-temperature and high-pressure adsorption of CO_2_, H_2_O, methanol, and dimethyl ether [75]. The sensor design is presented in Figure 7, which shows photographs of the coated and uncoated sensor and light microscopy images of the coated sensor. Water adsorption isotherms were measured at a range of pressures and temperatures (Figure 8). The determined adsorption parameters, i.e., adsorption capacities, adsorption enthalpies, and adsorption entropies, were found to compare well with the literature data.

**Figure 9:**
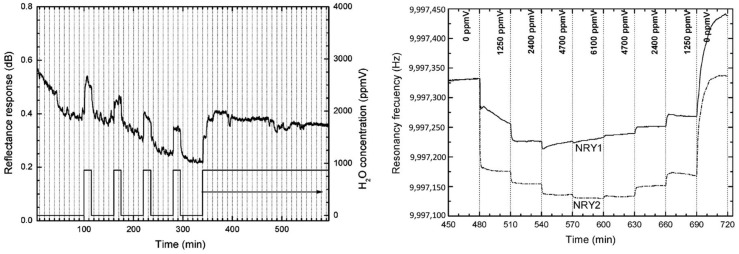
Evolution of reflectance response in NRQ-coated glass plates with water pulses of constant concentration in nitrogen (**left**). Evolution of frequency response in NRY1- and NRY2-coated QCM sensors upon introduction of increasing and decreasing concentrations of water (**right**) [76].

**Figure 10:**
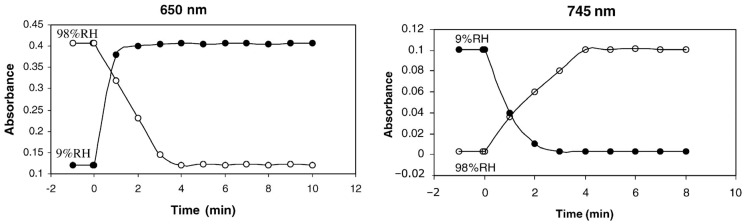
The response and recovery times for an MB-HMOR disc when relative humidity changes from low (9% RH) to high (98% RH) levels and the reverse. The results are shown for the measurements performed at both the 650 (**left**) and 745 nm (**right**) bands [66].

Zeolites have also been used as hosts for dyes in the development of optical detectors and sensors for humidity. For example, Pellejero et al. developed an optochemical sensor by encapsulating Nile Red in the supercages of NaY zeolite, denoted as NRY [76]. Figure 9 shows the reflectance response of such an optochemical sensor and the response of a QCM device covered with NRY. The Nile Red–Y composite exhibited excellent properties as a humidity sensor in terms of sensitivity (much lower than 200 ppm), response time (around 4 min), and dye stability toward migration upon high light exposures. Furthermore, the zeolite host provides an enhanced selectivity to water in mixtures with organic compounds. Indeed, the dye–zeolite composites were almost insensitive to *n*-hexane’s presence, regardless of the concentration, due to the selective adsorption imposed by the zeolite host. In another example, the protonation and deprotonation of methylene blue loaded into mordenite (MB-HMOR) demonstrated spectral changes over a relative humidity range of 9 to 98%. Figure 10 presents response and recovery times for an MB-HMOR disc when the relative humidity changes from low (9% RH) to high (98% RH) levels and the reverse. The system exhibited excellent reversibility, with rapid response times of approximately 2 min for adsorption and 4 min for desorption [66,77].

### 4.2. Zeolites as Sensors for Volatile Organic Compounds and Hydrocarbons

Volatile organic compounds (VOCs) are, as the name indicates, organic chemicals that have a high vapor pressure at ambient temperature. VOCs are used in various industries, having numerous toxic effects on the environment and human health. The severity of these effects depends on the concentration and exposure levels. The concentration of VOCs indoors can be 10 times higher than in the outdoor environment. Approximately 300 VOCs were identified in the air in schools, homes, offices, and various shopping malls and commercial buildings [78]. The limit of exposure that can cause death or delayed permanent adverse health effects has been defined by the National Institute of Occupational Safety and Health (NIOSH) and European Agency for Safety and Health at Work as immediately dangerous to life or health concentrations (IDLH) [79].

Among the most common VOCs are acetaldehyde, acetone, benzene, carbon tetrachloride, ethyl acetate, isopropyl alcohol, heptane, hexane, ethanol, naphthalene, formaldehyde, and toluene. Most of them are toxic, such as methanol, aromatic compounds, aldehydes, and ketones. Toluene, benzene, xylene, and ethyl-benzene are aromatic hydrocarbons that are recognized as the most harmful compounds among VOCs, with a high potential to affect human organ systems (e.g., circulatory, nervous, reproductive, cardiovascular, immune, and respiratory systems) [80]. Therefore, there is a need for effective sensory systems that can identify the presence of VOCs in a real time. Since, under real-life conditions, water is likely to be the most common interfering species, particularly in the case of polar VOCs (e.g., alcohols and aldehydes), zeolite-based sensors capable of detecting water and organic compounds are briefly considered first.

**Figure 11:**
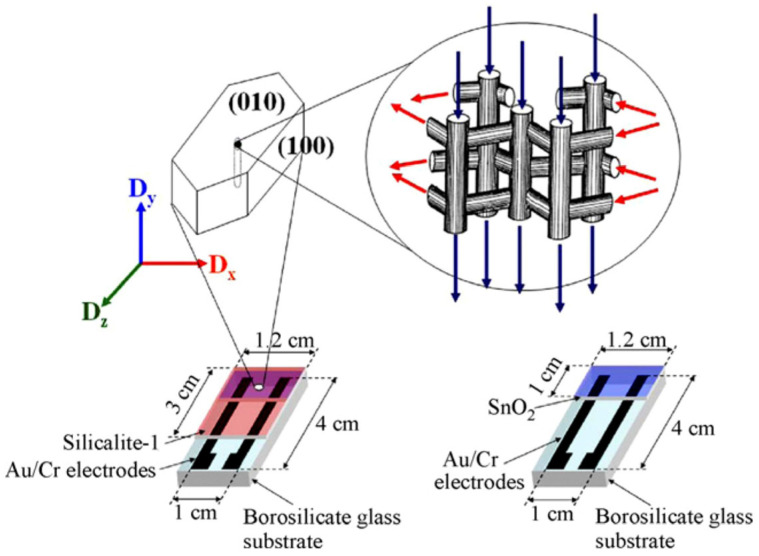
Schematic diagram of thin film sensors: (**right**) SnO_2_ thin film sensor and (**left**) Silicalite-1 coated on SnO_2_ thin film sensor [59].

**Figure 12:**
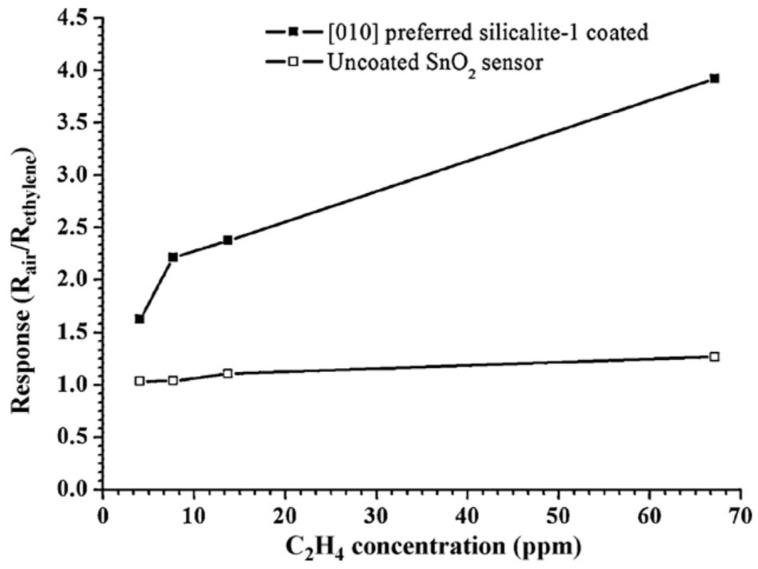
Dynamic range of ethylene detection for the uncoated SnO_2_ thin film sensor (☐) and the [010] preferred-orientation Silicalite-1 layer coated on the SnO_2_ thin film sensor (■) [59].

Conventional conductivity measurement methods have usually been used to detect VOCs. Sensors have been prepared using a Pd-doped SnO_2_ semiconductor with zeolites LTA and MFI as filters for the gas-phase sensing of different species (methane, propane, and ethanol) at different humidity levels [58]. The findings demonstrated that a zeolite layer significantly reduces, and in some instances eliminates, the sensitivity of the sensor to alkanes, thereby increasing its selectivity to alcohols. In another study, Silicalite-1 was deposited on SnO_2_ for ethylene sensing under humid conditions [59]. A schematic diagram of the sensor construction is presented in Figure 11. The zeolite acted as a shape-selective layer, which increased the hydrocarbon selectivity (Figure 12). This was attributed to the hydrophobic nature of Silicalite-1, which lacks aluminum atoms in its structure. The [010]-oriented zeolite layer exhibited improved response and recovery times (14 s and 144 s, respectively) compared to a randomly oriented layer (25 s and 208 s). The straight channels of Silicalite-1 were suggested to allow faster increases in ethylene concentration than randomly oriented crystallites. Sun et al. used an MFI-type zeolite with different SiO_2_/Al_2_O_3_ ratios and different grain sizes as a coating on a SnO_2_ semiconductor for formaldehyde and acetone sensing [81]. ZSM-5 (SiO_2_/Al_2_O_3_ = 70, grain size 300 nm)-coated SnO_2_ sensors exhibited an enhanced response to formaldehyde vapor, while the acetone response diminished in comparison with the SnO_2_ gas sensor. This result signified an improvement in the sensor’s selectivity. It should be noted here that the improvement in the sensitivity could be caused by the difference in kinetic diameters of the probed molecules in relation to the pore opening of the ZSM-5 (Figure 4). Formaldehyde (kinetic diameter 0.371 nm [82]) would exhibit little diffusion limitation, whereas acetone (0.616 nm [83]) will show impeded transfer through the zeolite pores. In contrast, the performance of the three ZSM-5 (with SiO_2_/Al_2_O_3_ ratios of 70, 150, and 470, respectively, and the grain size around 1 μm)-coated SnO_2_ sensors remained comparable to that of the SnO_2_ sensor. Therefore, it is clear that not only the pore size but also the grain size has an influence on the sensing performance. The larger crystallites could create films with larger cracks compared to smaller ones, allowing molecules free access to the SnO_2_ semiconductor. In a more recent publication, the researchers employed the electrospinning technique to achieve two objectives: to augment the interface area between materials and to mitigate the agglomeration of SnO_2_/ZSM-5 composite nanofibers [84]. The composite nanofibers exhibited a structure reminiscent of “bone joints,” featuring ZSM-5 nanoparticles. Subsequent analyses of their gas-sensing properties demonstrated that the selectivity and sensitivity of the sensors to formaldehyde underwent enhancement. Furthermore, the results of XPS analysis suggested that the adsorbed oxygen on the composite surface increased significantly, thereby enhancing the sensor sensitivity. Electrical equivalent circuit (EIC) analyses further indicated an increase in the electron transfer process due to an enlarged interfacial area between the zeolite and SnO_2_ composite. This outcome demonstrated an improvement in the sensitivity and the selectivity of the sensors. An interesting example of a VOC and hydrocarbon sensor utilized a Silicalite-1 film on a QCM (quartz crystal microbalance) for the detection of ethanol. Monitoring the adsorption of ethanol indicated a lower limit response at 50 ppm [85].

**Figure 13:**
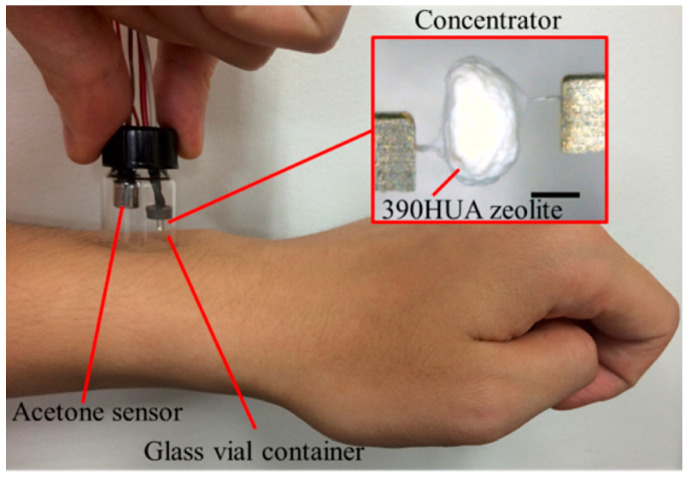
Photograph of skin acetone collection and measurement from human skin on the left forearm. A closed space is created using a glass vial containing the concentrator and acetone sensor. The scale bar corresponds to 500 μm [86].

**Figure 14:**
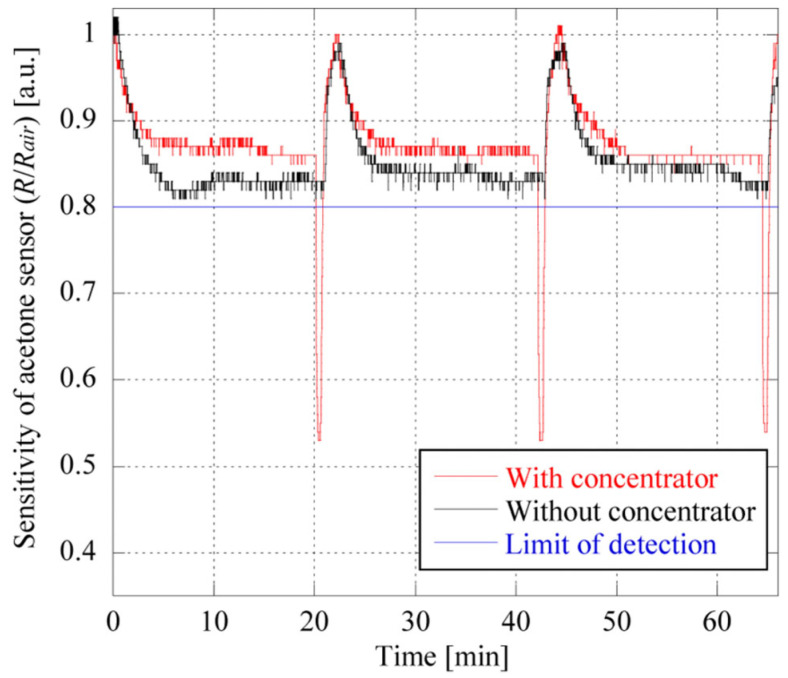
Continuous monitoring of the sensitivities of the semiconductor-based acetone sensor with and without the concentrator under simulated real conditions. The closed space was intentionally collapsed in a cyclic manner during each 20 min collection time. Acetone detection is possible when the sensitivity is less than 0.8 [86].

Zeolites can also be used to improve detection limits by concentrating analytes prior to detection. Yamada et al. investigated five different zeolites, including FER, MFI, and FAU, for concentrating skin acetone and subsequent semiconductor-based detection [86]. Figure 13 shows a device used for the collection and detection of acetone from human skin. A hydrophobic FAU zeolite (Si/Al = 250) with relatively large pores (0.74 nm, Figure 4, which is approximately 1.6 times larger than the acetone molecule) was the best “concentrator” of skin acetone among the zeolites tested. The device developed using zeolites was successfully applied in a semiconductor-based gas sensor in a simulated mobile environment, where the closed space was collapsed in a cyclic manner to reflect the twisting and elastic movement of skin that would be encountered in a wearable appliance (Figure 14). It was also demonstrated that the sensor could be regenerated at 330 °C using a coil heater.

**Figure 15:**
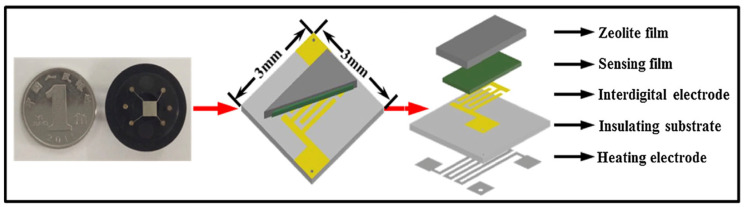
Schematic drawing and photograph of the sensor devices based on the configuration of zeolite films printed on top of sensing films [64].

**Figure 16:**
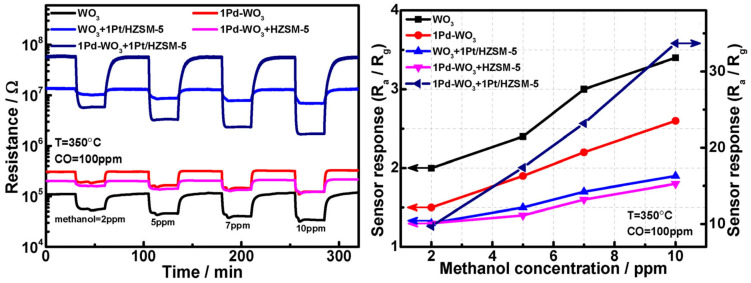
Responses of sensor created with Pt-loaded HZSM-5 films on Pd-WO_3_ to methanol in the presence of CO with a constant concentration of 100 ppm in dry air at 350 °C [64].

In a separate study, Zheng et al. evaluated gas sensors that were composed of Pd-loaded WO_3_ layers coated by zeolite films, with the objective of enhancing their selectivity towards methanol [64]. A schematic diagram and photograph of this sensor system are given in Figure 15. The incorporation of Pt-loaded HZSM-5 zeolite films into the Pd-WO_3_ sensor led to an enhancement in its sensitivity to methanol, with a detection limit as low as 0.5 ppm in the presence of high concentrations of CO. Figure 16 presents responses to methanol in the presence of CO with a constant concentration. The catalytic transformation of CO, facilitated by the Pt-modified HZSM-5 film, led to a p-type response to CO. The power-law relationship observed with oxygen showed a strong dependence of the resistive properties of the sensors on the oxygen concentration. This suggests that oxygen adsorbates on the sensor surface play a critical role in the fundamental sensing mechanism, similar to conventional gas sensors. The research concluded that the integration of zeolite films is a promising method for tailoring and improving the selectivity of gas sensors. In another study, sensors made of tungsten oxide and chromium titanium oxide coated with a layer of MFI or LTA demonstrated selective responses to ethanol and isopropanol vapors when operated at 400 °C [87]. This effect was attributed to a catalytic transformation of the analytes into species to which metal oxides provided differential responses.

Yimlamai et al. evaluated the electrical conductivity response of ZSM-5, MOR, and Y zeolites to ethanol [88]. The authors examined the influence of framework structure, charge-balancing cations (NH_4_⁺ or H⁺), and the hydrophilic/hydrophobic properties determined by the Si/Al ratio on detection. Sensitivity decreased with increasing Si/Al ratios, and zeolites in the NH_4_^+^ form showed negative responses, while all H⁺ forms displayed positive responses. Among the materials tested, HY with a Si/Al ratio of 30 demonstrated the highest sensitivity to ethanol vapor.

Thick films of Ca and Mg-STI (stilbite) were prepared via a screen printing process and then annealed at 650 °C and used as sensors for alcohols, such as ethanol and propanol [89]. It was shown that Mg-STI can sense ethanol and propanol at much lower temperatures as compared to a Ca-STI sensor. The ethanol uptake capacity of Mg-STI is higher than that of Ca-STI, whereas the propanol uptake capacities were the same for these sensors. Mg-STI showed a lower response time (~120 s) compared to Ca-STI (~300 s) for ethanol exposure, whereas Ca-STI gave a faster response (~135 s) to propanol compared to Mg-STI (~290 s). In a previous study, the molecular sieving of zeolite channels was evaluated by electrical impedance measurements with natural stilbite zeolite. This approach allowed the distinction of methanol, 2-propanol, and 3-pentanol from water and 2,2-dimethylpropanol [90]. The impedance of STI single crystals was dependent on the size, dipole moment, and the partial pressure of polar molecules in the gas phase. The interaction was described as purely adsorptive without catalytic oxidation and can be modeled by a Langmuir adsorption equation.

In a recent study, Joshi and colleagues modified a FAU-type zeolite with a Si/Al ratio of 1 by subjecting it to various temperatures (300–600 °C) in a 10% H_2_/90% N_2_ gas mixture. This treatment resulted in variations in the distribution of oxygen vacancies [91]. To characterize the presence of oxygen vacancies and electrons in the lattice, the researchers employed EDAX, XPS, and ESR techniques. The detection of various volatile organic compounds (VOCs), such as acetone, n-hexane, isopropyl alcohol, ethanol, ethyl methyl ketone, and benzene, was evaluated through the implementation of the scanning Kelvin probe (SKP) technique. The presence of oxygen vacancies was shown to enhance the distribution of both Lewis and Brønsted basic sites, thereby modifying the interaction of VOCs with the sample surface and the extent of VOC absorption. The investigation revealed that zeolites subjected to 600 °C exhibited n-type semiconductor characteristics. This treatment resulted in the presence of 13.8% oxygen-deficient sites, accompanied by 1.9 μmol/g of total basic sites and 3 μmol/g of total acidic sites. The findings demonstrated a ~280-fold enhancement in surface photovoltage towards acetone, with a recovery of ~67%.

Conductive polymers in conjunction with zeolites were demonstrated to be effective for VOC and hydrocarbon sensing. The K⁺ form of zeolite A, when combined with polypyrrole and polypyrrole polyamide-6, demonstrated sensitivity to methyl ether ketone, acetone, toluene, and methanol [92]. The fabrication method significantly influenced the findings: pellets and films showed vapor sensitivity, whereas electrospun fiber bundles showed no activity. For the sensing of methanol, the function of the zeolite was not fully elucidated, as the sensitivity of the composites could also be attributed to the interaction of methanol with polyamide-6. In the study by Permpool et al., a composite comprising dealuminated zeolite Y and nanoscale polydiphenylamine was utilized as a detection medium for distinguishing between various categories of solvent vapors, including non-polar, low-polarity, and high-polarity solvents [93].The investigation encompassed the exploration of the impact of surfactant composition, concentration, and the amount of dealuminated zeolite Y on the electrical conductivity, relative response, and selectivity of the composite. The composite with sodium dodecyl sulfate as a surfactant and 15% (*v*/*v*) dealuminated zeolite Y exhibited a comparatively elevated relative responsiveness to dichloromethane (DCM) vapors. The findings indicated that the composite’s response patterns to various solvents could be categorized as either non-polar or low polarity, yet this categorization was not applicable to high-polarity solvents. Furthermore, the interaction of the composite with DCM vapor was found to be reversible, as evidenced by cyclic response, FTIR, and electrostatic force microscopy images. When compared with standard microscale polydiphenylamine composites, the development of the nanoscale polydiphenylamine–dealuminated zeolite Y composite was identified as a promising sensor material for the detection of non-polar and low-polarity solvents.

Owing to their microporous catalytic properties, zeolites have been employed to the enhance selectivity toward specific analytes in gas mixtures. Screen-printed thick films of p-type SrTi_1−*x*_Fe*_x_*O_3−δ_ semiconductors were explored for the detection of hydrocarbons [94]. Formulations with 10% and 20% iron showed optimal performance among the tested compositions. The problem of the pronounced cross-interference of NO was successfully addressed by applying a zeolite layer of a different thickness and Pt content. The effect of the zeolite layer regarding NO cross-interference was explained by applying a simple diffusion–reaction model. Trimboli et al. incorporated platinum into zeolite Y and combined it with semiconducting TiO_2_ [95]. The resulting composite demonstrated selectivity for propane relative to CO. The authors proposed that propane oxidation, catalyzed by Pt-doped zeolite Y, generated water, which interacted with TiO_2_, leading to the observed changes in resistance.

Zeolite 4A was used for the fabrication of a capacitive sensor for monitoring dry air, N_2_, NO, and C_2_H_4_F_2_ [96]. The dielectric properties were influenced by the type and concentration of gas species in the environment. Significant capacitance changes were observed during dry air (+4.2%) and fluorinated gas (+7.3%) adsorption, whereas minimal dielectric variations were detected after exposure to N_2_ (−0.4%) and NO (−0.5%).

The Metglas–zeolite detector integrates the electromagnetic characteristics of a magnetoelastic material with the adsorption capabilities of porous crystalline materials. Gora et al. fabricated selective gas sensor by deposition of a b-oriented Silicalite-1 layer upon Metglas [97]. The device efficiently differentiated linear and branched hydrocarbons, i.e., it selectively detected *n*-butane, while it did not respond to the presence of *iso*-butane. This can be explained by the easier diffusion of *n*-butane (0.468 nm kinetic diameter [98]) in comparison to *iso*-butane (0.5278 nm [98]) in the pores of Silicalite-1 zeolite (Figure 4). It should be mentioned here that the concentrations of the targeted analytes were not specified. Therefore, the sensitivity and dynamic range of the device are unknown. In another study, an FAU–Metglas composite was used for the determination of six different VOCs [99]. At 120 °C and at 500 ppm, the sensor’s response decreased in the following order: ethyl-acetate > p-xylene > o-xylene ≈ benzene, c-hexane ≈ n-hexane. Furthermore, the minimum detection limits (MDLs) of c-hexane, p-xylene, and o-xylene were 166, 17, and 5 ppm respectively. In a later study, four zeolite films—FAU, LTA, MFI, and b-axis-oriented MFI—were prepared on magnetoelastic sensors. These films were intended to act as sensitive recognition films for the detection of cyclohexane, n-hexane, o- and p-xylene, and ethyl acetate [100]. The FAU sensor exhibited the minimum detection limits, down to 6 ppm for o-xylene, although it was not very selective. The LTA sensor demonstrated good sensitivity for the majority of hydrocarbons and showed selectivity towards xylene isomers. This was surprising given the small size of its micropores. Interestingly, the film with randomly oriented MFI showed the highest sensitivity of approximately 180 ppm to n-hexane in comparison to the other sensors. Furthermore, it displayed an opposite response to o-xylene and p-xylene compared to the b-oriented MFI.

Zeolites can also be used in optical sensing devices. Here, their exceptional adsorptive properties are used to increase the concentration of the analyte and therefore improve the sensitivity of the detector. For the determination of n-hexane, a 180 times-enhanced signal was observed when the ATR element was coated with Silicalite-1, as compared to an uncoated one [101]. Hugon et al. used two mordenite zeolites, with Si/Al ratios of 10 and 20, in combination with an integrated optical gas sensor for ethanol mixed with methane or *n*-hexane [102]. The selective determination of ethanol was achieved for both mixtures. Optical fibers coated with a zeolite thin film, which can interact with the analyte molecules to achieve high sensitivity for the real-time detection of trace organic vapors, have also been investigated. Ning et al. proposed a sensor comprised of the end face of a spherical fiber covered with a layer of zeolite thin film that constitutes an arc-shaped inline Fabry–Perot (F–P) cavity, which improves the interference performance [103]. The trace concentration of isopropanol vapor was measured by monitoring the shift in the F–P interference wavelength, which was induced by the adsorption of organic vapor by the zeolite film. The proposed sensor performed with an enhanced sensitivity of 91 pm/ppm, with a range from 0 to 70 ppm. A similar sensor was tested by Wu et al. for monitoring isopropanol and formaldehyde and their mixtures [33]. The experimental results showed that the sensitivities of the optical fiber sensor for monitoring isopropanol and formaldehyde were 281.9 pm/ppm and 4.99 pm/ppm, respectively. The optical fiber sensor was found more suitable for isopropanol measurement than for formaldehyde. In the mixed VOC state, the signal of the optical fiber sensor for isopropanol measurement was slightly changed when the sample chamber was exposed to a low concentration of formaldehyde. However, it should be noted here that in the articles published by Ning et al. [103] and by Wu et al. [33] the type of zeolite, which appeared to be Silicalite-1, was not specified, and no characterization details were presented.

The utility of zeolite materials for the selective detection of various VOCs is well documented in the scientific literature, and a summary of the quantitative data is provided in Table 1. The minimum concentrations detected for the organic compounds vary significantly, from 2 ppm to thousands of ppm. Optical and resistive sensors have been showing the best potential for the development of detection systems, which in the future should be tested using the concept of an “electronic nose”, that is, an array of sensors that is used to identify a number of pollutants simultaneously and in real time. This is particularly important as there is an apparent lack of competitive detection studies of target analytes from mixtures of potentially interfering chemicals. Pollutants in real-life systems occur in mixtures with complex components and in wide concentration ranges, and selective detection from mixtures is significantly more challenging and should be the focus of future studies. A further issue is that in many of the reviewed articles, insufficient characterization details are provided for the crucial properties of the zeolites used in the sensors. In some cases, it is not clear which zeolite structure was used; in others basic characterization data, such as XRD, surface area, Si/Al ratio, TEM, and SEM, are missing, or the data are contradictory. Zeolites are complex materials, and several factors need to be considered to understand their mechanisms of interaction with VOC molecules and their sensing, in order to design sensitive and selective detectors. Therefore, there is a need for greater collaborative and interdisciplinary effort to advance this area of science.

## 5. Conclusions and Outlook

Zeolites offer considerable flexibility and versatility in terms of selectivity and sensitivity, thereby demonstrating significant potential for the fabrication of sensors for a range of gases and vapors. Zeolite-based detectors exhibit both size and shape selectivity induced by the dimensions of their pores, enabling the detection of and differentiation between gases and vapors. The incorporation of select dopants into their frameworks has been shown to control chemical selectivity and sensitivity, eliciting desirable optical or electrochemical responses upon interaction with analytes.

However, zeolites also show significant drawbacks that still hinder their sensing application. In specific applications, zeolites may exhibit diminished charge carrier transport, which can result in a reduced signal quality relative to inorganic metal oxide gas sensors. This can impact the sensitivity and reliability of the sensor’s readings. Furthermore, zeolite-based gas sensors can demonstrate a delayed response to certain compounds due to factors such as suppression effects, selective adsorption, and diffusion behaviors, which can be influenced by the type of zeolite and its interaction with the target gas. Additionally, zeolite sensors can experience cross-sensitivity to water vapor. This can obscure the detection of target molecules, especially in environments with varying humidity levels. Although this interference can be reduced, the effectiveness depends on the specific arrangement and hydrophilicity of the zeolite used.

One of the possible ways to improve on the low conductivity of zeolites in sensing applications is the design of 2D material–zeolite composite materials. Since the successful exfoliation of 2D graphene in 2004, the family of 2D nanomaterials has made remarkable progress, expanding into various multifunctional applications. The unique atomic-layered configuration of 2D materials confers various advantages, such as an extremely large specific surface area that nears the theoretical maximum, elevated carrier mobility, substantial adsorption capabilities for analyte molecules, and increased surface reactivity. These characteristics confer a fundamental advantage upon 2D materials in gas-sensing applications, as their electronic properties are markedly responsive to the adsorption of chemical analytes. Furthermore, the formation of heterostructures is possible, whereby the electrical transport capacity can be modified by varying the thickness or band gap of the 2D semiconductor, which presents the potential to creatively manipulate the sensing capabilities of 2D materials. However, their limitations in terms of incomplete recovery, insufficient sensitivity at low detection limits, limited gas selectivity, and susceptibility to humidity at room temperature have curtailed their practical utility in various applications. One of the most promising methods for enhancing the stability and selectivity of two-dimensional (2D)-based sensors is through the incorporation of porous materials, such as zeolites. By a careful selection of materials and the mode of combining them, the disadvantages of both types of materials—the low stability and selectivity of 2D materials on the one hand, and the low conductivity of zeolites on the other hand—could be effectively overcome.

Despite their numerous advantages, zeolites also display diffusion limitations for bulkier molecules with a size similar to the size of thire micropores, which in sensing application leads to a delayed response to certain compounds. A fundamental approach to minimizing diffusion limitations is to reduce the diffusion path length. This can be achieved by developing hierarchically porous materials that integrate porosities of multiple levels in a single solid body. Hierarchical zeolites have been utilized with great success in the fields of heterogeneous catalysis and adsorption. Zeolites with different hierarchically porous structures, i.e., a micro-mesoporous structure, micro-macroporous structure, and micro-meso-macroporous structure, can be prepared and tuned for the timely detection of specific analytes in a mixture of gases.

## Figures and Tables

**Table 1:** Zeolite materials reported for VOC sensing with descriptions of the sensor types and the concentration ranges that were investigated.

Sensing Material	Analytes	Detector/Sensor Type	Dynamic Range * (Where Stated)	Ref.
MFI, LTA/Pd/SnO_2_	Ethanol	Resistive	10–50 ppm	[58]
Silicalite-1/SnO_2_	Ethylene	Resistive	5–70 ppm	[59]
ZSM-5/SnO_2_	Formaldehyde	Resistive	2–100 ppm	[84]
Silicalite-1	Ethanol	QCM	50–700 ppm	[85]
Pt-HZSM-5/Pt-WO_3_	Methanol	Resistive	2–30 ppm	[32]
Mg-stilbite	Ethanol	Resistive	100–1000 ppm	[89]
Zeolite 3A/polypyrrole, polyamide	Methanol	Resistive	1–6%	[92]
Zeolite Y/polydiphenylamine	DCM, Acetone	Resistive	1000–12,000 ppm	[93]
Pt-ZSM-5/SrTi_1−*x*_Fe*_x_*O_δ−ı_	Propane	Resistive	150–2000 ppm	[94]
Zeolite Y/Pt-TiO_2_	Propane	Resistive	250–1000 ppm	[95]
Zeolite 4A	1,1-Difluoroethane	Capacitive	100–500 ppm	[96]
Silicalite-1	n-hexane	Infrared	60 ppm	[101]
Mordenite	n-hexane	Optical fiber	8000 ppm	[102]
Silicalite-1	Isopropanol	Optical fiber	0–70 ppm	[103]
Silicalite-1	Isopropanol, formaldehyde	Optical fiber	(CH_3_)_2_CHOH: 0–70 ppm CH_2_O: 0–100	[33]

* Lower value in the dynamic range corresponds to the minimum concentration that was tested.

## Data Availability

The original contributions presented in the study are included in the article; further inquiries can be directed to the corresponding authors.
